# Supplementary material for: MetaQTL: a package of new computational methods for the meta-analysis of QTL mapping experiments
Source: BMC Bioinformatics. 2007 Feb 8;8:49. doi: 10.1186/1471-2105-8-49 (PMC1808479; doi:10.1186/1471-2105-8-49)
Supplement: Additional File 7 — MetaQTL Package : jar file and tutorial. This Zip archive contains both the MetaQTL JAR file and the files of the tutorial. [file 1471-2105-8-49-S7.zip › org.inra.metaqtl/doc/org/metaqtl/adapter/MetaMapAdapter.html]

MetaMapAdapter


|  |  |  |
| --- | --- | --- |
| **Overview** \| **Package** \| **Class** \| **Use** \| **Tree** \| **Deprecated** \| **Index** \| **Help** | |  |
| **PREV CLASS**   **NEXT CLASS** | **FRAMES**    **NO FRAMES**     **All Classes** |  |
| SUMMARY: NESTED \| FIELD \| CONSTR \| METHOD | DETAIL: FIELD \| CONSTR \| METHOD |  |


---


## org.metaqtl.adapter Class MetaMapAdapter

```
java.lang.Object
  org.metaqtl.adapter.MetaMapAdapter
```

---

``` public final class MetaMapAdapter extends java.lang.Object ```

This class defines some usefull methods to adapt `MetaMap`
objects into other kinds of objects or the inverse.

---

**Constructor Summary**

`MetaMapAdapter()`


| **Method Summary** | |
| --- | --- |
| `static IBioGenome` | `toIBioGenome(MetaMap metaMap)`             This methos convert a `MetaMap` object into an object which is consistent with the `IBioGenome` interface. |

**Methods inherited from class java.lang.Object**

`clone, equals, finalize, getClass, hashCode, notify, notifyAll, toString, wait, wait, wait`

**Constructor Detail**

### MetaMapAdapter

```
public MetaMapAdapter()
```


**Method Detail**

### toIBioGenome

```
public static IBioGenome toIBioGenome(MetaMap metaMap)
```

:   This methos convert a `MetaMap` object into
    an object which is consistent with the `IBioGenome`
    interface.

    :   **Parameters:**: `metaMap` - the `MetaMap` to convert. **Returns:**: an object which class implements the `IBioGenome` interface.


---


|  |  |  |
| --- | --- | --- |
| **Overview** \| **Package** \| **Class** \| **Use** \| **Tree** \| **Deprecated** \| **Index** \| **Help** | |  |
| **PREV CLASS**   **NEXT CLASS** | **FRAMES**    **NO FRAMES**     **All Classes** |  |
| SUMMARY: NESTED \| FIELD \| CONSTR \| METHOD | DETAIL: FIELD \| CONSTR \| METHOD |  |


---
